# The Elusive Endometrial Epithelial Stem/Progenitor Cells

**DOI:** 10.3389/fcell.2021.640319

**Published:** 2021-04-09

**Authors:** Fiona L. Cousins, Ronald Pandoy, Shiying Jin, Caroline E. Gargett

**Affiliations:** ^1^The Ritchie Centre, Hudson Institute of Medical Research, Clayton, VIC, Australia; ^2^Department of Obstetrics and Gynecology, Monash University, Clayton, VIC, Australia; ^3^Buck Institute for Research on Aging, Novato, CA, United States

**Keywords:** endometrium, adul stem cell, progenitor cell, epithelial cells, stem cell niche, lineage tracing, human, mouse

## Abstract

The human endometrium undergoes approximately 450 cycles of proliferation, differentiation, shedding and regeneration over a woman’s reproductive lifetime. The regenerative capacity of the endometrium is attributed to stem/progenitor cells residing in the basalis layer of the tissue. Mesenchymal stem cells have been extensively studied in the endometrium, whereas endometrial epithelial stem/progenitor cells have remained more elusive. This review details the discovery of human and mouse endometrial epithelial stem/progenitor cells. It highlights recent significant developments identifying putative markers of these epithelial stem/progenitor cells that reveal their *in vivo* identity, location in both human and mouse endometrium, raising common but also different viewpoints. The review also outlines the techniques used to identify epithelial stem/progenitor cells, specifically *in vitro* functional assays and *in vivo* lineage tracing. We will also discuss their known interactions and hierarchy and known roles in endometrial dynamics across the menstrual or estrous cycle including re-epithelialization at menses and regeneration of the tissue during the proliferative phase. We also detail their potential role in endometrial proliferative disorders such as endometriosis.

## Introduction

### The Endometrium

The endometrium is a unique tissue that undergoes monthly cycles of proliferation, differentiation, breakdown, shedding and repair under the control of fluctuations in circulating ovarian hormones, 17 β-estradiol and progesterone (Jabbour et al., 2006). The endometrium is composed of two layers. The basalis, adjacent to the myometrium, is not shed at menstruation and from this layer the functionalis arises each month (Gargett et al., 2012). The functionalis, the upper layer of the endometrium, undergoes the most structural changes during the menstrual cycle. During the proliferative phase, under the influence of ovarian-derived estradiol (Ferenczy et al., 1979; Punyadeera et al., 2006), the endometrial glandular epithelium, stroma, and vasculature undergo extensive proliferation. Three dimensional (3D) reconstruction reveals basalis glands form horizontal, branching networks, whereas the functionalis glands grow vertically from these branches (Tempest et al., 2020; Yamaguchi et al., 2020). During the secretory phase, under the influence of ovarian-derived progesterone, the functionalis undergoes changes to prepare for pregnancy; the endometrial epithelial cells differentiate into secretory cells, producing histotroph to nourish an implanting embryo (Burton et al., 2002), regions of stromal cells differentiate into epithelial-like decidual cells, spiral arterioles remodel and uterine natural killer cells become the dominant leukocyte to assist with allorecognition (Gibson et al., 2015). In the absence of a pregnancy, the corpus luteum regresses, circulating progesterone concentrations fall and the functionalis loses structural integrity and sheds in a piecemeal fashion (Garry et al., 2009).

### Regenerative Capacity of the Endometrium

**FIGURE 1:**
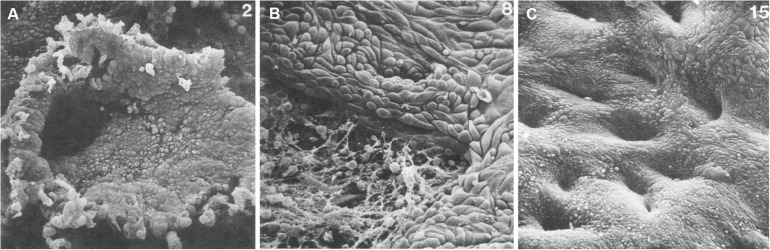
Scanning electron microscopy of human endometrial microarchitecture during menstruation. **(A)** Day 2, basalis glandular epithelial stump protrudes into the uterine lumen. **(B)** Day 4, newly formed luminal epithelium progressively covering fibrin-coated denuded areas. **(C)** Day 7, re-epithelialization has been completed. Adapted with permission from Ludwig and Spornitz (1991).

Whilst the outward manifestation of menstruation, vaginal bleeding, may be experienced for 5 days or longer in some women, repair processes are initiated at the beginning of this process. Immediate post-menstrual repair involving re-epithelization of the luminal epithelium commences within 48 h of onset (Ferenczy, 1976) in a steroid hormone-depleted micro-environment, and when epithelial estrogen receptor alpha (ERα, ESR1) expression is low (Okulicz and Scarrell, 1998; Figure 1). Indeed, estrogen is not required for endometrial re-epithelialization as evidenced in animal models of endometrial repair (Matsuura-Sawada et al., 2005; Kaitu’u-Lino et al., 2007). Histological and scanning electron microscopic examination of menstrual phase endometrium reveals epithelial extensions of glandular epithelium over the denuded surface (Ferenczy, 1976; Ludwig and Spornitz, 1991), supporting the concept that new luminal epithelial cells arise from the residual basal glandular epithelium (Figures 1B,C). Mesenchymal to epithelial transition (MET) may also occur during re-epithelialization (Garry et al., 2010; Patterson et al., 2013; Cousins et al., 2014), where residual stromal fibroblasts undergo cellular transformation to form new luminal epithelial cells. However, a recent cell fate tracing study using multiple Cre-loxP activated models found no evidence of MET in cycling adult endometrium (see section on the role of endometrial epithelial stem/progenitor cells in re-epithelization and post-partum regeneration).

**FIGURE 2:**
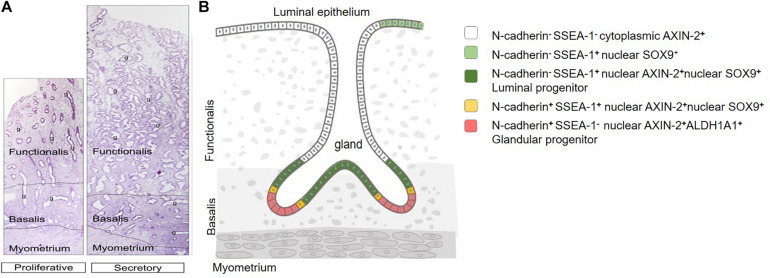
Human endometrial epithelial location and hierarchy. **(A)** Full-thickness proliferative and secretory stage premenopausal endometrium. Functionalis and basalis delineated by dotted line. Glands (g) extend from the luminal epithelium to the endometrial-myometrial junction, showing branching and horizontal gland profiles in the deep basalis. **(B)** Epithelial stem/progenitor hierarchy. Adapted with permission from Gargett et al. (2012) and Filby et al. (2020).

Post menstruation, the functionalis endometrium grows from the remaining basalis which has a thickness of 0.5 mm, reaching a maximum thickness of 7–8 mm by the mid-proliferative phase (McLennan and Rydell, 1965; Figure 2A). This remarkable regenerative capacity is likely mediated by stem/progenitor cells located in the basalis layer (Chan et al., 2004; Gargett, 2007a). Different populations of endometrial stem/progenitor cells have been identified, including endometrial mesenchymal stem cells (eMSCs) and endometrial epithelial stem/progenitor cells (eES/PCs). The focus of this review are the eES/PCs. Readers interested in eMSCs are referred to a recent detailed review (Bozorgmehr et al., 2020).

### Cyclical Turnover in the Mouse Endometrium

**FIGURE 3:**
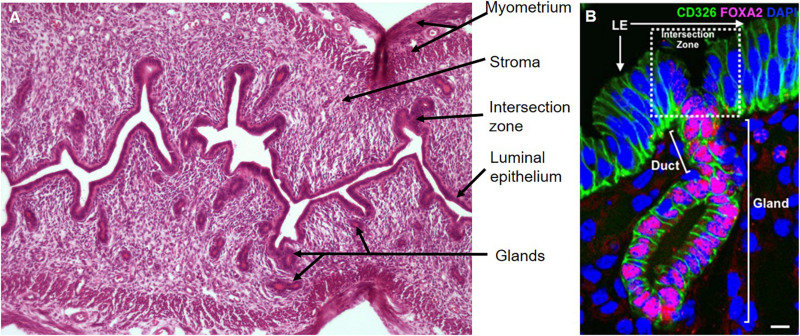
Mouse endometrium and epithelial unit. **(A)** Longitudinal section of estrous cycling endometrium, hematoxylin, and eosin stained. **(B)** A representative uterine epithelial unit stained with CD326 (EpCAM, epithelial marker, green) is composed of LE, duct and single gland labeled by FOXA2 (red) in adult wild-type uterine tissue section. The dotted line shows the intersection zone between luminal and gland epithelial compartments. Scale bar: 5 μm.

Similar to human, the endometrial epithelium of the adult mouse uterus consists of luminal (LE) and glandular (GE) epithelia, two histologically and functionally distinct cell types (Figure 3A). The simple columnar LE lines the inner surface of the endometrium, the cuboidal GE forms tubular gland structures surrounded by stromal cells. Like women, the mouse endometrium responds to cyclical changes in circulating ovarian steroid hormones, but they do not menstruate. Whereas women undergo an approximate 28 days menstrual cycle, mice have an estrous cycle lasting approximately 4 days (Nelson et al., 1982). The estrous cycle comprises four stages; the estrogen-dominated proestrus and estrus stages and the progesterone-dominant metestrus and diestrus (Byers et al., 2012). Unlike women, the mouse endometrium does not undergo spontaneous decidualization in the presence of progesterone, it requires a physical stimulus, i.e., the presence of a blastocyst, for a decidual reaction to occur (Finn, 1977). Similar to human endometrium, the mouse endometrium is composed of a myometrium, a thin compact basal layer and a loosely compacted functional stromal layer covered with a luminal epithelium and glands penetrating the stromal layer to form a mucosa.

During proestrus, under the influence of increasing concentrations of circulating estrogens, uterine water content, height of LE cells and GE proliferation increases (Wood et al., 2007). During oestrus, the uterus is distended, and the endometrial glands exhibit maximal secretory activity (Bertolin and Murphy, 2014). In the absence of pregnancy, the endometrium enters metestrus, where degeneration occurs, the LE and GE undergo significant apoptosis (Wood et al., 2007) and the LE undergoes vacuolar degeneration (Bertolin and Murphy, 2014). As the endometrium enters diestrus, LE cells are columnar and endometrial glands are atrophic in the absence of steroidal support (Bertolin and Murphy, 2014).

Menstruation can be mimicked in a mouse using exogenous steroids and inducing artificial decidualization via delivery of sesame oil into the lumen of the uterine cavity (Brasted et al., 2003; Cousins et al., 2014). Post-partum repair can also be modeled in mice using pseudopregnancy models (Fan et al., 2008; Rudolph et al., 2012; Patterson et al., 2013) to provide a useful model for studying endometrial dynamics.

Whilst the cyclical changes in the mouse endometrium during the estrous cycle are not as dynamic as in human endometrium, putative stem/progenitor populations have been identified in the mouse endometrium, which likely support cell turnover and endometrial repair and regeneration post-partum (Chan and Gargett, 2006; Huang et al., 2012; Cao et al., 2015). While definitive endometrial epithelial stem/progenitor cell markers are still lacking for both mouse and human, new markers of these cells are emerging, which will enable their role in endometrial regeneration to be determined in the near future.

## Identifying Epithelial Stem/Progenitor Cells

### Characterization of Adult Stem Cells

Stem/progenitor cells are rare cells present in most postnatal tissues and organs, where they function in maintaining cellular homeostasis of the tissue or organ (Snyder and Loring, 2005; Gargett, 2007b). Stem/progenitor cells are initially identified by their functional attributes that distinguish them from the bulk of the cells comprising the tissue or organ. Identifying criteria of stem/progenitor cells are self-renewal, high proliferative potential and capacity to differentiate into one or more cell types of the tissue in which they reside (Potten and Loeffler, 1990). Paradoxically, epithelial stem/progenitor cells are quiescent and rarely proliferate, despite their ability to initiate a cascade of daughter cell proliferation to restore tissue homeostasis following tissue damage. The stem cell niche, comprising the stem/progenitor cell and neighboring differentiated niche cells, secreted molecules and extracellular matrix regulates resident stem/progenitor cell proliferation and cell fate decisions (Eckfeldt et al., 2005).

### Functional Assays of Stem/Progenitor Cell Activity

Initially, human stem/progenitor cells are characterized by functional assays assessing their key attributes as there are no universal stem/progenitor cell markers. Clonogenicity, defined as the ability of a single cell to initiate a colony of cells when seeded as single cells at extremely low seeding densities, is the most commonly used approach for identifying a stem/progenitor cell activity (Gargett, 2007a). Self-renewal is a defining feature of stem/progenitor cells and can be assessed by serial cloning of individual cells *in vitro* (Gargett et al., 2009) and in serial transplantation at limiting cell numbers *in vivo* (Asselin-Labat et al., 2006). Proliferative potential is assessed by serial passaging of cells to calculate the number of population doublings before senescence is reached (Li et al., 1998; Gargett et al., 2009). Differentiation is determined by culture of the putative stem/progenitor cell population in induction media containing key differentiation factors or transplanting them into orthotopic or ectopic sites (e.g., kidney capsule) and analyzing the cells formed in the neo-tissue generated (Kaur et al., 2004; Joseph and Morrison, 2005).

Although there is no universal marker that defines the many human stem/progenitor cell types identified to date, some are common for several of these cells from different tissues. Some stem/progenitor cell markers have functional roles in tissue homeostasis, but often this is not the case. Some markers may be a stem/progenitor cell marker in one tissue, e.g., CD34 is a hemopoietic stem cell marker in bone marrow, but also marks mature endothelial cells in other tissues. It is important that any phenotypic marker defining a specific stem/progenitor cell population has been verified to enrich for the cell type in one or more of the functional assays listed above (Kaur et al., 2004; Gargett, 2007a). Side population (SP) cells, identified as a small population of cells capable of effluxing the vital DNA-binding dye, Hoechst 33342 by dual wavelength flow cytometry, may be used as an assay of potential stem/progenitor cells in a cell population (Challen and Little, 2006). Another approach uses label retention of DNA synthesis labels, such as bromodeoxyuridine (BrdU), in studies which may indirectly predict potential stem/progenitor cell populations retaining the label following a chase period while their proliferating progeny rapidly dilute the label to histologically non-detectable levels (Braun and Watt, 2004). Further evidence is required to functionally verify the stem or progenitor identity of cells labeled by both approaches.

### Lineage Tracing to Identify Stem/Progenitor Cells

Lineage tracing is a powerful technique used to identify stem/progenitor cells. It has evolved since its initial use in the late nineteenth century, where dyes and fluorescent tracers were the most commonly used approach (Kretzschmar and Watt, 2012). Using a pulse-chase approach, a single marked cell is traced for a length of time by following the transmission of the cell’s mark to its progeny. Analyzing the cellular phenotype, location, and number of the marked progeny, provides information on the identity of the initial marked cell that generated a clone of cells *in vivo*. Using this technique, researchers have extensively identified adult stem cell populations in the intestine (Barker et al., 2007), liver (Wang et al., 2015), and uterus (Jin, 2019).

Today, the predominant method of lineage tracing is cell marking by genetic recombination. Here, the expression of a cell or tissue specific recombinase enzyme leads to the subsequent expression of a conditional reporter gene. This allows for the permanent genetic labeling of a cell and its future progeny. There are two widely used recombination systems: one adapted from bacteriophage P1 (Cre-loxP) and used predominantly in mice, and the other adapted from *Saccharomyces cerevisiae* (FLP-FRT) and used predominantly in Drosophila. This review will focus on the Cre-loxP recombination system used in mice. In this site-specific recombination system, one mouse line contains a tissue or cell-specific promotor expressing Cre recombinase, with the enzyme’s activity temporally controlled through its fusion with an estrogen receptor (Feil et al., 1997) or progesterone receptor (Kyrkanides et al., 2003). Activation of Cre recombinase is dependent on the administration of tamoxifen or mifepristone, estrogen and progesterone receptor-binding ligands, respectively (Feil et al., 1997; Kyrkanides et al., 2003). This Cre recombinase containing mouse line is crossed with a mouse line containing a reporter gene, such as *Rosa26-lacZ*, *Rosa26-GFP*, *Rosa26-tdTomato* flanked by a loxP-STOP-loxP sequence. The administration of tamoxifen or mifepristone results in the tissue or cell-specific activation of Cre recombinase and the enzyme’s excision of the STOP sequence, allowing expression of the reporter gene (β*-galactosidase*, *GFP, tdTomato* for the above examples, respectively), and the permanent genetic labeling of the tissue or cell population and their progeny. The use of low ligand doses allows for the labeling of individual cells and their clones, with subsequent lineage tracing potentially leading to the identification of a stem/progenitor cell population.

Self-renewal and differentiation, hallmarks of stem cells, can be directly assessed by single-cell lineage tracing under physiological conditions. In one approach, a single cell is genetically marked to enable transmission of that mark to the cell’s progeny, resulting in a labeled clone. The properties of the labeled clone determine whether or not it is a stem cell clone, thus identifying the founder cell as stem cell or not (Fox et al., 2009). The lineage mark does not change the properties of the marked cell, or its progeny, or the surrounding environment (Kretzschmar and Watt, 2012). Thus, lineage tracing reflects a cell’s physiological behavior and fate in the context of its stem cell niche in the intact tissue, which is not possible in non-niche environments, such as *in vitro* clonogenicity assays or transplantation. Another advantage of single-cell lineage tracing is that it can be performed in any cell type without knowing the specific gene markers of this cell type (Kretzschmar and Watt, 2012). Using the Cre-loxP recombination system in mice has led to numerous discoveries of stem cell populations. Intestinal epithelial stem cells were discovered using the marker gene leucine-rich repeat-containing G-protein coupled receptor 5 (*Lgr5*) (Barker et al., 2007). Initial screening identified *Lgr5* as a *Wnt* target with expression restricted to the intestinal crypts. A transgenic mouse line containing a knock-in fluorescently tagged *Lgr5* promotor next to an inducible Cre recombinase and the *Rosa26-lacZ* reporter strain was used to trace the lineage of *Lgr5*^+^ cells over time. Observation and quantification of the number of clones, their position, and differentiated clonal cell phenotypes identified *Lgr5*^+^ crypt base columnar cells as the epithelial stem cells of the intestine (Barker et al., 2007). Similarly, using the *Wnt*-responsive marker gene *Axin2*, an epithelial stem cell population was discovered in mouse liver (Wang et al., 2015). Using a mouse line containing an *Axin2* promotor positioned next to an inducible Cre recombinase and the *Rosa26-mTmG* reporter strain, the expression of *Axin2*^+^ cells was traced, showing they produced clones that expanded concentrically from the central veins. The pericentral *Axin2*^+^ cells were capable of self-renewal and differentiating into the hepatocyte population (Wang et al., 2015).

## Human Endometrial Epithelial Stem/Progenitor Cells

Endometrial epithelial stem/progenitor cells were first identified as clonogenic cells, comprising 0.22% of single cell suspensions of EpCAM^+^ epithelial cells obtained from hysterectomy tissue which includes the basalis layer (Chan et al., 2004; Schwab et al., 2005). Both large (0.08% of epithelial cells) and small clones (0.14%) were generated. The frequency of clonogenic human endometrial epithelial cells using limiting dilution analysis was 1/174 (0.57%) epithelial cells (Gargett et al., 2009), similar to epithelial colony forming unit (CFU) cells. In serum-free medium, stromal feeder layers and growth factors EGF, TGFα, or PDGF-BB were required for growth indicating the importance of epithelial-stromal interaction. Large endometrial epithelial clones underwent self-renewal *in vitro* as demonstrated by serial cloning at very low seeding densities (10–20 cells/cm^2^) (Gargett et al., 2009), 35–45 population doublings and differentiation into large gland like structures in 3D organoid-type cultures. In comparison, small epithelial clones showed limited self-renewal, proliferation and only generated small spheroidal structures. Endometrial SP cells are heterogeneous and include all cell lineages of human endometrium, of which 27% are EpCAM^+^ epithelial cells (Miyazaki et al., 2012; Gargett et al., 2016). SP cells very occasionally reconstitute epithelial glands (0.02–8%) when transplanted into immunocompromised mice (Masuda et al., 2010; Cervelló et al., 2011). While these attributes of rare epithelial cells indicate stem/progenitor cell activity in human endometrium they provide no evidence of their location or stem cell niche.

### Markers and Location

#### Human Endometrial Basalis Epithelial Markers

It has been hypothesized that human endometrial epithelial stem/progenitor cells are located in the basalis layer, thereby providing a source of cells to regenerate the endometrial functionalis each month (Gargett, 2004, 2007b; Figure 2B). Thus, initial attempts to find specific markers for these stem/progenitor cells focused on the basalis layer. The first basalis-specific epithelial marker identified was nuclear AXIN2 in 2012. *AXIN2* was discovered using a gene microarray approach, comparing highly purified EpCAM^+^ epithelial cells isolated from pre- and post-menopausal hysterectomy endometrium (Nguyen et al., 2012). The rationale for this approach was based on the following assumptions; post-menopausal endometrial epithelial cells have a similar gene expression profile to basalis epithelial cells of pre-menopausal endometrium, that the functionalis would dilute gene expression of the basalis in pre-menopausal endometrium, and that estrogen stimulates the scant epithelial cells present in atrophic post-menopausal endometrium to regenerate functionalis-like glands. Indeed, the gene profile of post-menopausal endometrial epithelium showed marked similarity to laser-captured micro-dissected menstrual endometrial epithelium (Gaide Chevronnay et al., 2009). Many WNT signaling pathway molecules were differentially expressed, with *AXIN2* and *SOX9* upregulated in post-menopausal endometrial epithelial cells. Immunofluorescence and confocal microscopy showed specific nuclear AXIN2 immunoreactivity in pre-menopausal basalis epithelial cells, while cytoplasmic AXIN2 was observed in functionalis epithelium (Nguyen et al., 2012). *AXIN2* mRNA and nuclear SOX9 and β-catenin proteins have since been described in basalis glands of human endometrium (Valentijn et al., 2013; Syed et al., 2020). As nuclear markers, AXIN2, SOX9 and β-catenin are not convenient markers for prospective isolation of basalis epithelial cells to demonstrate stem/progenitor cell functional activity. Surface markers are required.

#### Surface Markers of Human Endometrial Epithelial Stem/Progenitor Cells

Two surface markers have been identified in subpopulations of basalisepithelium that enrich for stem/progenitor cell activity in several functional assays confirming stem/progenitor status. One is N-cadherin, identified in an unbiased approach using the same pre- versus post-menopausal gene profiling of highly purified EpCAM^+^ epithelial cells from hysterectomy tissue described in the previous section (Nguyen et al., 2017). Of the 11 surface markers showing increased expression in post-menopausal epithelial cells, *CDH2* was the most consistently differentially expressed as shown in the heat map and confirmed by qPCR in a validating set of endometrial epithelial samples (Nguyen et al., 2017). Importantly, N-cadherin^+^ (protein encoded by the *CDH2* gene) endometrial epithelial cells were more clonogenic than N-cadherin^–^ epithelial cells, showed greater self-renewal and more population doublings by serial cloning. They also differentiated into cytokeratin-expressing organoids in 3D culture. N-cadherin-immunostained epithelial cells were located in the bases of the glands adjacent to the myometrium in pre- and post-menopausal endometrium (Nguyen et al., 2017). They colocalized with cytokeratin, ERα and E-cadherin, suggesting they were not undergoing epithelial-mesenchymal transition (EMT). They were generally quiescent as few immunolocalized with the proliferation marker, KI67. N-cadherin^+^ cells were localized to the apical and lateral surfaces of the epithelial cells and rarely colocalized with the basalis epithelial marker nuclear SOX9. It is possible that the N-cadherin^+^ cells are located on horizontal branching and rhizome like glandular structures recently identified in the basalis of human endometrium (Tempest et al., 2020; Yamaguchi et al., 2020).

A second marker, SSEA-1 or CD15, identifies basalis epithelial cells in pre-menopausal and post-menopausal endometrium (Valentijn et al., 2013). While the stem/progenitor cell activity of freshly isolated SSEA-1^+^ epithelial cells has not yet been determined, cultured SSEA-1^+^ cells form larger spheroids in 3D cultures than SSEA^–^ cells. SSEA-1^+^ cells have longer telomeres and greater telomerase activity than SSEA-1^–^ cells, characteristics suggestive of a stem/progenitor cell. Cultured SSEA-1^+^ spheroids show weak immunoreactivity for nuclear ERα or PR, suggesting they were derived from the ill-defined basalis-functionalis junction rather than the deep basalis, since basalis epithelial cells express ERα throughout the menstrual cycle (Leyendecker et al., 2002). Of interest is that nuclear SOX9 is found in SSEA-1^+^ cells and some SSEA-1^+^ cells show nuclear β-catenin, indicating active WNT signaling, potentially maintaining an undifferentiated epithelial state. Co-localization with another WNT signaling marker, nuclear AXIN2 protein has not yet been reported for SSEA-1^+^ cells. The luminal epithelium also contains numerous SSEA-1^+^ cells with nuclear SOX9. Whether these have stem/progenitor function remains uncertain given that they are shed each month during menstruation (Valentijn et al., 2013) but they could be derived from the glandular epithelial cells that re-epithelialize the denuded surface during menstruation, although there is no proof (Figure 1).

Another marker, LGR5, is a receptor for R-spondin and functions in the canonical WNT signaling pathway. LGR5 is a surface marker of intestinal epithelial stem cells, but is a controversial marker of human endometrial epithelial stem/progenitor cells, as evidenced by conflicting reports of its expression during the menstrual cycle (Gil-Sanchis et al., 2013; Tempest et al., 2018a), most likely due to the poor quality of available antibodies and co-localization with leukocyte markers CD45 and CD163 (Tempest et al., 2018a). Human LGR5^+^ endometrial epithelial cells have not been assessed in functional stem cell assays during the normal menstrual cycle or in post-partum regeneration, limiting our understanding of the identity of these cells. Organoid culture of LGR5^+^ epithelial cells would be beneficial in assessing the self-renewal and differentiation properties of human LGR5^+^ endometrial epithelial cells. It is clear further work is required to validate LGR5^+^ as a definitive endometrial epithelial stem/progenitor cell marker.

A range of stem cell markers in non-endometrial tissues have been investigated in human endometrium, but their validation as markers of epithelial cells with stem/progenitor activity has not been determined. These have been summarized in a recent review (Tempest et al., 2018b).

### Endometrial Epithelial Stem/Progenitor Cell Hierarchy

Dual color immunofluorescence of N-cadherin with SSEA-1 or SOX9 in human endometrium showed little co-localization by confocal microscopy (Nguyen et al., 2017). Rather, SSEA-1^+^ and SOX9^+^ epithelial cells were proximal to N-cadherin^+^ epithelial cells and appeared to overlap the basalis-functionalis “junction”, suggesting a potential differentiation hierarchy of epithelial cells exists in human endometrial epithelium (Figure 2B). The most primitive may be the clonogenic, self-renewing N-cadherin^+^SSEA-1^–^ epithelial cells located in the deepest gland profiles, some of which appear to branch or are only found on half a profile (Nguyen et al., 2017). These may give rise to a very small population of N-cadherin^+^SSEA-1^+^ cells closer to the functionalis, which in turn generate the more proximal N-cadherin^–^SSEA-1^+^ epithelial cells which appear to span the basalis-functionalis “junction” and are also present in the luminal epithelium. The most numerous and most differentiated epithelial cells are N-cadherin^–^SSEA-1^–^ cells of the functionalis glands. N-cadherin also colocalizes with the ALDH1A1 isoform of an epithelial stem cell lineage marker, aldehyde dehydrogenase I (ALDH1), in the deep basalis, with 78% of N-cadherin^+^ cells showing colocalization (Ma et al., 2020). This potentially suggests additional cell types in the endometrial epithelial stem/progenitor cell hierarchy. ALDH1A1 is a cytoplasmic enzyme that converts retinal to retinoic acid suggesting that the retinoic acid pathway may have important roles in clonogenic N-cadherin^+^ cells. The distribution of ALDH1A1 suggests these cells are unlikely to co-express SSEA-1 or SOX9. Recently, single cell transcriptomics of human endometrial biopsy tissue showed a small distinct population of ciliated epithelial cells (Wang et al., 2020). However, neither *CDH2*, *ALDH1A1* nor *FUT4* [α-(1,3)-fucosyltransferase 4] catalyzing protein glycosylation associated with the expression of SSEA-1 were identified in the gene profiles, suggesting the basalis epithelium was not sampled. It is also not known whether ciliated cells are part of the putative endometrial epithelial hierarchy. Nor is it clear how this epithelial hierarchy are located in 3D endometrium, given the degree of gland branching and rhizome formation in the basalis glands (Tempest et al., 2020; Yamaguchi et al., 2020).

Mutations in cytochrome c oxidase (CCO) have been used to visualize cell lineages in the intestine (Taylor et al., 2003). In the endometrium, *in vivo* lineage tracing using mitochondrial DNA passenger mutations as clonal markers identified a stem cell niche in the basalis GE (Tempest et al., 2020). Multiple CCO-deficient cell clusters are located in the basalis GE and genome sequencing of each cluster revealed common somatic mutations, indicative of a similar cell of origin (Tempest et al., 2020). Individual glands in the functionalis may have more than one epithelial stem/progenitor cell as they appear to arise from horizontal branching glands. Interestingly, the number of CCO-deficient clusters increased with age, peaking at age 50 before declining around age 60, indicative of stem quiescence associated with menopause (Tempest et al., 2020). Reassessing the apparent epithelial hierarchy in horizontal branching basalis glands and in the vertical glands that appear to sprout from them will be important. It also explains why only the basal half of some gland profiles contained N-cadherin^+^ epithelial cells (Nguyen et al., 2017). This horizontal glandular structure of the deepest endometrial glands suggests a mechanism that prevents their shedding during menstruation, thus preserving a glandular reservoir of stem/progenitor cells required for regenerating the functionalis glands each month (Tempest et al., 2020).

The current model of the putative stem/progenitor cell populations in human endometrium are based on 2D imaging with its inherent limitations. Future research using *in vivo* lineage tracing of passenger mutations (as above), new tissue clearing methods, slice cultures (see below), organoid models and molecular (sequencing) will enable investigation of the epithelial hierarchy in 3D and at the single cell level. These approaches will inform and may build on the current models.

### Role of Stem/Progenitor Cells in Endometrial Repair and Re-epithelialization

Endometrial repair following menstruation is a rapid process that occurs over a 48-h period in the absence of circulating estrogens. Scanning electron microscopy studies show that repair is initiated on days 2–3 of the menstrual cycle and is completed by days 4–5 (Ferenczy, 1976) as evidenced by an intact luminal epithelium. The endometrium sheds and repairs concurrently in a piecemeal fashion, as shown by areas of new luminal epithelial cells adjacent to shedding functionalis (Garry et al., 2009). There are a number of potential mechanisms of re-epithelialization, the most commonly accepted theory suggesting that the new luminal epithelium arises from the glandular epithelium of exposed basalis glands (Novak and Te Linde, 1924). These cells migrate from the protruding stumps of glands over the denuded surface to rapidly form a new luminal epithelium (Ludwig and Spornitz, 1991; Figure 1). This mechanism likely explains the presence of luminal epithelial SSEA-1^+^SOX9^+^ cells described above, as the functionalis layer regenerates from the re-epithelialized basalis, and the luminal epithelium retains the SSEA-1^+^SOX9^+^ phenotype as it is pushed ever upwards during endometrial growth. It is also possible that residual SSEA-1^+^ luminal epithelial cells may be activated at menstruation to support rapid re-epithelialization during piecemeal shedding of the functionalis, given the adhesive and migratory properties of SSEA-1^+^ cells (Valentijn et al., 2013). Shedding functionalis remnants can also get trapped under the new migrating luminal epithelium, where they are reorganized and incorporated into the newly developing functionalis in the subsequent cycle (Henriet et al., 2012). This may also explain the presence of SSEA-1^+^ cells in the new luminal epithelium.

Another proposed mechanism is MET, where stromal cells close to the luminal surface appear to become new luminal epithelial cells (Garry et al., 2010). These cells can be identified by dual staining of mesenchymal and epithelial markers, such as cytokeratin and vimentin. As a mesodermal-derived epithelium, human endometrial epithelium co-expresses cytokeratin and vimentin. Whether these cells are derived from an endometrial progenitor population, such as a basalis epithelial progenitor that has previously undergone EMT remains to be elucidated. MET has been studied more comprehensively in mouse models of regeneration, which are described below.

### Role of Epithelial Stem/Progenitor Cells in Endometrial Regeneration

The contribution of stem/progenitor cells in endometrial regeneration has been well documented in xenograft models, where single cell suspensions of endometrial epithelial and stromal cells transplanted under the kidney capsule self-organize into endometrial glands and stroma (Masuda et al., 2007). These endometrial like structures respond to cyclical exogenous estradiol and progesterone and exhibit blood-filled cyst formation when steroid hormone support is withdrawn (Masuda et al., 2007). Clonally-derived side population cells of epithelial and stromal origin can also form endometrial-like structures under the kidney capsule (Cervelló et al., 2011).

A new *in vitro* system involving human endometrial tissue slice culture shows promise in enabling the investigation of the role of epithelial progenitor cells in endometrial regeneration. Of particular importance is that this culture system provides a multicellular, 3D “*in vivo*-like” system, which maintains endometrial zonation (Muruganandan et al., 2020). In this model, tissue slices respond to estrogen and progesterone over a 21-day period. LacZ staining via adeno-mediated gene delivery can be achieved, however, specific delivery to only the epithelium needs further optimization (Muruganandan et al., 2020). This model has the potential for investigating the interactions and dynamics of epithelial stem/progenitor cells *in situ*, particularly during the estrogen dominant proliferative phase of rapid endometrial functionalis growth.

As described earlier, a potential epithelial progenitor hierarchy exists in the glandular epithelium, which is thought to support regeneration of the tissue as estradiol concentrations begin to rise following menstruation. N-cadherin^+^ cells express ERα (Nguyen et al., 2017) and, as is typical for stem/progenitor populations, rarely proliferate. As in other tissues, such as the intestine, more mature cells in the endometrial epithelial hierarchy may be responsible for glandular epithelial cell proliferation in the rapidly growing functionalis glands. Such cells, defined as a transit amplifying population, with the capacity to rapidly proliferate and produce more differentiated cells, amplify the output from each stem cell division. These transit amplifying cells (TAC) are present either in the functionalis as an ERα^+^ TAC or possibly as SSEA-1^+^ TAC around the basalis-functionalis junction.

Epithelial expression of SOX9 is higher in the proliferative phase than the secretory phase (Saegusa et al., 2012) and co-localizes with SSEA-1 in the basalis epithelium. It has been suggested that SOX9 may act as a checkpoint to prevent hyperplasia (Prévostel et al., 2016) highlighting its importance in the epithelial cell regulation and overall tissue homeostasis. Since SOX9 is a WNT pathway transcription factor and the WNT/β catenin pathway is critical to maintaining epithelial cell integrity in other organs, such as the intestine (Fevr et al., 2007), it is possible that SOX9 plays a key role in epithelial cell proliferation following menstruation. Nuclear AXIN2 is expressed by basalis epithelial cells (Nguyen et al., 2012) in both pre- and post-menopausal women, where it acts as a negative regulator of WNT signaling to maintain the epithelial stem/progenitor niche (Nguyen et al., 2012). Clearly more detailed studies at the single cell level are needed to delineate the roles of the various cells of the endometrial epithelial hierarchy in endometrial re-epithelialization and regeneration.

### Role of Stem/Progenitor Cells in Endometrial Pathologies—Endometriosis and Endometrial Cancer

Endometriosis is characterized by the presence of endometrial-like tissue in the peritoneal cavity. Retrograde menstruation, where menstrual fragments flow backward through the fallopian tubes into the peritoneal cavity, is likely the main cause of endometriosis. Given that 90% of all women exhibit retrograde menstruation, and the prevalence of endometrial cells in the peritoneal cavity is similar in women with or without the disease (Dorien et al., 2017), other mechanisms must be involved to account for the subset of women who develop endometriosis. Whilst the number of endometrial cells in the peritoneal fluid does not differ, the cell types contained in the shed tissue may have an important role. Indeed, it has been hypothesized that endometrial epithelial stem/progenitor cells are shed in menstrual fluid which gain access to the peritoneal cavity by retrograde menstruation where they initiate lesions via their clonogenic activity (Gargett, 2007b; Gargett et al., 2016; Cousins et al., 2018a; Filby et al., 2020). Women with endometriosis have more SSEA-1^+^SOX9^+^ epithelial cells in their functionalis compared to normal women. These cells can form 3D structures *in vitro*, suggesting that they may generate lesions *in vivo* when the functionalis is retrogradely shed at menstruation (Hapangama et al., 2019). Similar to healthy controls, the eutopic expression of LGR5 does not change over the menstrual cycle. However, an increase in the expression of LGR5 was observed in ectopic lesions when compared to eutopic endometrium (Vallvé-Juanico et al., 2018) which may suggest its involvement in disease pathogenesis. Menstrual effluent of women with endometriosis also contains an increased number of basalis fragments (Leyendecker et al., 2002), suggesting that the resident stem/progenitor cell populations may also contribute to the survival of tissue fragments reaching the pelvic cavity.

Endometrial epithelial stem/progenitor cells have rarely been isolated from menstrual blood, although endometrial mesenchymal stem cells and stromal fibroblasts are well characterized in menstrual fluid (Musina et al., 2008; Bozorgmehr et al., 2020). The endometrial epithelial basalis marker SSEA-1 has been identified in ectopic endometriosis lesions (Valentijn et al., 2013) which may support their role in lesion establishment and progression (Valentijn et al., 2013). SOX9, a marker of stem/progenitor activity in other tissues, is normally expressed in the basalis, but women with endometriosis exhibit a higher number of SSEA-1^+^SOX9^+^ cells in the functionalis during the secretory phase of the menstrual cycle. Isolated SSEA-1^+^SOX9^+^ cells differentiated into endometriotic gland like structures in 3D culture (Hapangama et al., 2019). Deep basalis epithelial markers ALDH1 isoforms ALDH1A1, and ALDH1A3 are increased in the epithelium of ovarian endometriomas, and ALDH1A3 is increased in the epithelium of lesions found on the bowel (Ma et al., 2020), potentially suggesting the cells were derived from basalis epithelium. All of these findings suggest that endometriosis lesion survival depends on the presence of one or more basalis-derived epithelial stem/progenitor cells.

Endometrial cancer is the most common gynecological cancer. Cancer stem cells (CSCs) are implicated in tumor initiation, progression, metastasis and recurrence. Endometrial CSCs are thought to originate through several mechanisms including; genetic mutation or epigenetic alteration of epithelial stem/progenitor cells residing in the tissue, de-differentiation of endometrial epithelial cells which form a CSC progenitor, or via EMT of endometrial Side Population cells (Giannone et al., 2019). Endometrial CSC were initially identified as clonogenic cells which generated tumors recapitulating the histology and several markers of the parent tumors when transplanted in limiting dilution into an immunocompromised mouse model (Hubbard et al., 2009). The tumors could be serially transplanted indicating self-renewal of the tumor-initiating cells. Putative epithelial stem/progenitor marker CD44 has been suggested as an endometrial CSC marker, showing upregulation in endometrial carcinoma compared to normal endometrium (Gao et al., 2012; Torres et al., 2019). SOX9 is up-regulated in endometrial cancer and in endometrial hyperplasia (Gonzalez et al., 2016). N-cadherin protein is also increased in the glandular epithelium of endometrioid adenocarcinomas (Xie et al., 2017), highlighting a role for abnormal basalis-derived epithelial stem/progenitor cells in endometrial proliferative diseases.

## Mouse Endometrial Epithelial Stem/Progenitor Cells

At birth, the murine uterus lacks endometrial glands and consists of a tube lined with a simple luminal epithelium supported by undifferentiated mesenchyme. The LE forms buds which invade the mesenchyme to initiate the development of GE around post-natal day 5 (P5). Around P7, histologically distinct uterine glands appear in the endometrium (Branham et al., 1985; Gargett and Chan, 2006), which continue to extend from the LE into the surrounding endometrial stroma forming the basic adult configuration of the murine uterus by P15 (Gray et al., 2001). Individual uterine epithelial units, comprising a region of LE, glands surrounded by stromal cells and the intersection zone between LE and the gland (Figure 3B) form the basic structure of the entire endometrial epithelium (Jin, 2019). The endometrium becomes functional, undergoing cyclical regression and regeneration, when the reproductive hormones estrogen and progesterone are secreted by the ovaries. The LE regulates embryo attachment for implantation, and GE regulates embryo survival and growth, stromal cell decidualization and placental development (Wang et al., 2013; Spencer et al., 2019; Ye, 2020). To date, there are no definitive stem cell markers for the mouse endometrium.

### Location of Murine Endometrial Epithelial Stem/Progenitor Cells

Before more sophisticated methods were available, DNA label retention was used extensively to predict the existence and location of potential endometrial epithelial stem/progenitor cells. Epithelial label retaining cells (LRCs) are absent or very rare after a 3- to 4-week chase in postnatal and prepubertal mouse models and predominantly found in the LE along with rare LRC in the GE (Gargett and Chan, 2006; Cervelló et al., 2007; Patterson and Pru, 2013). These LE LRCs do not express Esr1 (ERα), in contrast to neighboring Esr1^+^ non-LRC. This molecular difference may be used to characterize their identity and function (Gargett and Chan, 2006; Chan et al., 2012). LRCs initiated estrogen-induced endometrial epithelial regeneration in ovariectomized mice (Chan et al., 2012). By applying genetic labeling of H2B-GFP, peripubertal labeling resulted in glandular LRCs persisting for 8 months and through several pregnancies (Patterson and Pru, 2013). However, long-term persistent glandular LRCs were not seen post H2B-GFP labeling in adult cycling mice (Wang et al., 2012). Thus, the LRC approach is limited in definitively identifying stem/progenitor populations and their location, likely due to variables such as timing of the initial pulse, length of chase and labeling cells on their penultimate cell division (Gargett et al., 2016).

Mouse telomerase reverse transcriptase (mTert) marks stem cells in the intestine (Breault et al., 2008) and was recently shown to mark rare stromal, epithelial and leukocyte populations in the mouse endometrium. Epithelial mTert expression does not co-localize with BrdU, indicating that mTert is independent of LRCs and marks a different progenitor cell type (Deane et al., 2016). Wild-type recipients of bone marrow transplants from *mTert-GFP* or *Ch*β*-actin-GFP* reporter mice demonstrated no contribution of bone marrow-derived cells to endometrial epithelial lineages, but contributed to immune cells which were likely misidentified in previous studies (Ong et al., 2018).

The first *CreERT2-LoxP*–based single-cell lineage tracing system in the adult mouse uterus to functionally identify epithelial stem cells resulted from characterizing stem cell clones *in vivo* (Jin, 2019). In this study, a mouse line containing a *Keratin19* (epithelial marker) promotor positioned next to an inducible Cre recombinase was crossed with the *Rosa26-YFP* reporter strain to lineage label epithelial cells. By quantifying distinct cellular phenotypes (EpCAM^+^FOXA2^–^ luminal vs. EpCAM^+^FOXA2^+^ glandular) and the proliferative ability (KI67^+^) of the endometrial epithelium, different clonal populations were identified in the mouse endometrial epithelium. The founder cells of mixed clones, originating from the intersection zone of the LE and GE, were identified as endometrial epithelial stem cells (Jin, 2019; Figure 3B). Such tissue distribution supports that these bipotent endometrial epithelial stem cells bidirectionally differentiate into LE and GE for maintaining homeostasis and regeneration of mouse endometrial epithelium under physiological conditions (Jin, 2019).

Another recent lineage tracing study in the mouse uterus claimed *Axin2*-expressing cells residing in endometrial glands as the stem cell source responsible for epithelial regeneration (Syed et al., 2020). In this study, tetracycline induction and Cre-mediated recombination system were combined to label and trace the behavior and fate of the *Axin2*-expressing cells. Around 29% of GE are *Axin2*^+^, querying the enrichment level of the endometrial epithelial stem cell population, which is expected to be rare as for other adult stem cells. Lineage tracing *Axin2*^+^ cells revealed their location in gland bases, where they progressively expanded to occupy entire glands after 90 days using an initial subset of labeled *Axin2*^+^ GE cells, or after 70 days when all *Axin2*^+^ GE cells were labeled and traced. The mouse endometrium is a highly regenerative tissue with substantial epithelial turnover during each 4-to 5-day estrous cycle. A 90-day trace is equivalent to 22–23 cycles, and 70 days equates to 14–15 cycles. However, there was a limited contribution of *Axin2*^+^ GE to the LE under both experimental conditions during normal estrous cycling. Even after six cycles of pregnancy and involution (180 days) following 1 week of maximally labeled *Axin2*^+^ GE cells, the contribution of *Axin2*^+^ GE to the LE is minimal. Thus, *Axin2*^+^ GE has a very limited contribution to LE after multiple cycling or post-partum injury, but a high contribution to GE, suggesting *Axin2*^+^ GE supply to LE is insufficient to maintain homeostasis or renewal of LE (Syed et al., 2020). It appears that *Axin2*^+^ GE is a GE-specific progenitor cell, particularly given that the cellular turnover of LE is substantially greater than GE in cycling mice (Kaitu’u-Lino et al., 2010). Using a different GE specific marker, lineage tracing of *Foxa2*^+^ GE fate completely excluded the contribution of GE to LE (Jin, 2019). This was further verified by evidence that *Lgr5*^+^ progenitor cells located on the tips of developing endometrial glands after birth are exclusively responsible for the development and maintenance of uterine glands (Seishima et al., 2019). Lineage tracing of *Pax8*^+^ epithelial cells revealed a potential cellular source to maintain both luminal and glandular epithelia, however, their potency is difficult to be determined, as *Pax8* is abundant throughout the entire endometrial epithelium (Fu et al., 2020). The epithelial stem cell population located in the intersection area between LE and GE maintains and renews both LE and GE efficiently to supply cellular requirement during cycling and the post-partum period (Jin, 2019). Thus, the intersectional location of the adult endometrial epithelial stem cells well supports the physiology and function of mouse uterine endometrium.

The niche for endometrial epithelial stem cells in both mice and humans is not as well studied as other organs. Endometrial stem/progenitor cells are a relatively new field and it took time to identify specific marker (s) for these cell populations in human and they are still to be identified in the mouse. This limits the identification of their microenvironment on a cellular and molecular level. There is currently insufficient published knowledge to provide a detailed understanding of the endometrial epithelial stem cell niche in this review. However, it is anticipated that new studies in the next few years will generate discoveries on the endometrial epithelial stem cell niche.

### Role of Endometrial Epithelial Stem/Progenitor Cells in Re-epithelization and Post-partum Regeneration

Stem cell contributions to re-epithelization can be studied using mouse models of menstruation (Brasted et al., 2003; Cousins et al., 2014) or pseudopregnancy (Huang et al., 2012; Patterson et al., 2013). MET has been studied in both models. Cells expressing both cytokeratin and vimentin were observed close to areas of repair within 12 h of progesterone withdrawal in a menses-like model (Cousins et al., 2014), with significant increases of epithelial *Wnt7a* mRNA coinciding with decreasing concentrations of stromal *Wnt4* mRNA suggesting stem/progenitor activity and MET. In a postpartum repair model, a group of stromal cells expressing Anti Mullerian Hormone Receptor type II contributed to epithelial repair and regeneration via MET (Huang et al., 2012; Patterson et al., 2013). A comprehensive investigation into a role for MET in endometrial regeneration using a number of different lineage tracing mouse lines indicates that mesenchymal reporter-positive epithelial cells were identified at birth and maintained in adult epithelium, as expected for a mesodermal-derived epithelium. However, evidence of MET of adult mesenchymal cells, particularly during post-partum repair, was not identified suggesting that it is unlikely that the mesenchyme contributes to the adult epithelium (Ghosh et al., 2020).

SP cells have been found in the stroma in postpartum mice but not in the normal cycling endometrium (Hu et al., 2010). The identity and role of endometrial SP cells remain unclear. Unlike in estrous cycling mice, mTert^+^ LE cells were not observed prior to endometrial breakdown, most likely due to the high exogenous progesterone support. Following progesterone withdrawal and induction of menses, LE mTert^+^ clusters were identified in the repairing epithelium (Cousins et al., 2018b), suggesting activation to support immediate repair mechanisms. During this initial repair event no GE mTert^+^ cells are identified, suggesting that GE expression of mTert may be estrogen dependent and may have a role in endometrial regeneration. LE mTert^+^ cells were located adjacent to clusters of KI67^+^ cells suggesting that mTert^+^ cells may undergo asymmetrical division to form TACs, which proliferate to form new LE cells (Cousins et al., 2018b). Similarly, extensive cell turnover of the LE was demonstrated during the repair phase in an induced menstruation-like event, which was followed by GE proliferation (Kaitu’u-Lino et al., 2010). These concepts are also in keeping with how the GE supports regeneration in the human endometrium.

Whilst the bone marrow does not appear to contribute to the endometrial epithelium during the estrous cycle, bone marrow transplantation studies under pathological conditions have revealed a limited contribution of bone marrow-derived cells to endometrial regeneration (Bratincsak et al., 2007; Du and Taylor, 2007; Mints et al., 2008; Du et al., 2012; Morelli et al., 2013).

Lineage tracing has provided direct evidence that the epithelial stem cells in the intersection zone between LE and GE are capable of life-long maintenance of the self-renewing endometrial epithelium and post-partum regeneration of epithelial lineages (Jin, 2019). *Axin2*^+^ (Syed et al., 2020) or *Lgr5*^+^ (Seishima et al., 2019) progenitor GE cells located at the tips (base) of glands support the cyclical renewal and/or post-partum regeneration of endometrial GE in mice. It would be interesting to reveal the relationship among the epithelial stem cells in the intersection area, *Axin2*^+^ and *Lgr5*^+^ GE progenitors in future studies. It is possible that the epithelial stem cells in the intersection area differentiate into either *Axin2*^+^ or *Lgr5*^+^ GE progenitors with significant overlap between these 2 progenitor cells given the role of both markers in the *Wnt* signaling pathway.

## Conclusion

Accumulating reports of endometrial epithelial stem/progenitor cells have revealed their important roles in homeostasis and regeneration of the endometrial lining of the uterus in both humans and mice. Increasing knowledge of endometrial stem/progenitor cell biology and their niches provides new understanding of the remarkable regenerative capacity of mouse and human endometrium. It also contributes new insight into endometrial proliferative disorders, offering potential for new therapies targeting the epithelial stem/progenitor cells. In mice, lineage tracing single cells in the whole uterus reliably tracks the behavior and fate of the endometrial epithelial stem/progenitor cells, by which, potency, location and markers of endometrial epithelial stem/progenitor cells have been advanced. In humans, previous and recent studies applying functional stem cell assays including organoid formation and specific surface marker identification have enabled characterization of the location of endometrial epithelial stem/progenitor cells. The surface markers identified for human endometrial epithelial stem/progenitor cells allow their isolation and future manipulation for treatment of infertility or miscarriage caused by an inadequate endometrial proliferation. Single cell sequencing comparing basalis, functionalis and luminal epithelium will likely increase our understanding of the epithelial hierarchy in human endometrium in relation to the newly discovered unique structural differences between these endometrial zones. The regulatory mechanisms of self-renewal and differentiation of endometrial epithelial stem/progenitor cells are the scientific premise needed to decode aberrations in these cells and their role in the development of endometrial diseases such as endometriosis and endometrial cancer. Only then can effective treatments be developed that target abnormal endometrial epithelial stem/progenitor cells. The promise of endometrial epithelial stem/progenitor cells for regenerative medicine, their markers and their regulatory mechanisms of self-renewal and differentiation should ensure further research in these areas are pursued.

## Author Contributions

All authors listed have made a substantial, direct and intellectual contribution to the work, and approved it for publication.

## Conflict of Interest

The authors declare that the research was conducted in the absence of any commercial or financial relationships that could be construed as a potential conflict of interest.
